# COVID-19 and risk of neurodegenerative disorders: A Mendelian randomization study

**DOI:** 10.1038/s41398-022-02052-3

**Published:** 2022-07-14

**Authors:** Chunyu Li, Jiayan Liu, Junyu Lin, Huifang Shang

**Affiliations:** 1 Department of Neurology, Laboratory of Neurodegenerative Disorders, National Clinical Research Center for Geriatrics, West China Hospital, Sichuan University, Chengdu, Sichuan China; 2 Department of Dermatology and Venerology, West China Hospital, Sichuan University, Chengdu, Sichuan China

**Keywords:** Genetics, Scientific community

## Abstract

Emerging evidence has suggested a close correlation between COVID-19 and neurodegenerative disorders. However, whether there exists a causal association and the effect direction remains unknown. To examine the causative role of COVID-19 in the risk of neurodegenerative disorders, we estimated their genetic correlation, and then conducted a two-sample Mendelian randomization analysis using summary statistics from genome-wide association studies of susceptibility, hospitalization, and severity of COVID-19, as well as six major neurodegenerative disorders including Alzheimer’s disease (AD), amyotrophic lateral sclerosis, frontotemporal dementia, Lewy body dementia, multiple sclerosis, and Parkinson’s disease. We identified a significant and positive genetic correlation between hospitalization of COVID-19 and AD (genetic correlation: 0.23, *P* = 8.36E–07). Meanwhile, hospitalization of COVID-19 was significantly associated with a higher risk of AD (OR: 1.02, 95% CI: 1.01–1.03, *P*: 1.19E–03). Consistently, susceptibility (OR: 1.05, 95% CI: 1.01–1.09, *P*: 9.30E–03) and severity (OR: 1.01, 95% CI: 1.00–1.02, *P*: 0.012) of COVID-19 were nominally associated with higher risk of AD. The results were robust under all sensitivity analyses. These results demonstrated that COVID-19 could increase the risk of AD. Future development of preventive or therapeutic interventions could attach importance to this to alleviate the complications of COVID-19.

## Introduction

Coronavirus disease 2019 (COVID-19), caused by the severe acute respiratory syndrome coronavirus 2 (SARS-CoV-2), has spread across the world and led to substantial morbidity and mortality [1]. Due to the multifactorial pathogenesis of COVID-19, various complications have been observed in patients with COVID-19 discharged from hospitals, such as fatigue, impaired pulmonary function, kidney injury, and neurological manifestations [2, 3]. However, some long-term consequences might not be observed promptly, especially in observational studies which might be biased by unavoidable confounding factors. Identifying potential complications of COVID-19 might help better understand the pathogenesis of this epidemic, and facilitate therapeutic options which could alleviate the complications of COVID-19.

Recent findings have shown a close correlation between COVID-19 and neurodegenerative characteristics [4, 5], bringing the potential role of COVID-19 in the future development of neurodegenerative diseases into the spotlight. Neurodegenerative disorders are characterized by a slow progressive loss of neurons in the central nervous system (CNS), which leads to deficits in specific brain functions. It is becoming clear that COVID-19 can affect CNS, and patients diagnosed with COVID-19 may develop neurological symptoms [6]. From the epidemiological perspective, previous retrospective analysis of over 200,000 patients in the UK found that 1.74 and 0.26% of patients with intensive therapy unit (ITU) admission due to COVID-19 developed dementia and parkinsonism respectively in the 6 months after initial infection [5]. Meanwhile, neurodegenerative biomarkers like neurofilament light chain (NfL) and glial fibrillary acidic protein (GFAP) were higher in COVID-19 patients than non-COVID-19 patients with mild cognitive impairment or Alzheimer’s disease (AD), and these markers were correlated with the severity of COVID-19 [4]. Pathologically, SARS-CoV-2 spike enters host cells by binding to its receptor human ACE2 (hACE2) through its receptor-binding domain (RBD) [7]. Direct SARS-CoV-2 viral invasion of the CNS occurs in a subset of patients with COVID-19 [8], and SARS-CoV-2 might infect brain cells and damage neurons, thus affecting CNS and triggering neurological symptoms [9, 10]. Previous study has shown that SARS-CoV-2 could infect neural tissues and cause significant neuronal death based on experimental evidence in human brain organoids, mice with over-expressing ACE2, and autopsies from patients who died of COVID-19 [10]. Meanwhile, the innate immune responses and cytokine storm triggered by COVID-19 might also promote the development or progression of neurodegeneration [11]. Peripherally released cytokines could cross the blood-brain barrier, thus causing direct neurotoxicity and contributing to the activation of microglia and astrocytes [12]. Meanwhile, peripheral immune cells could participate in the progression of neuroinflammatory and neurodegenerative diseases by infiltrating the brain [13]. Patients with severe COVID-19 infection have been reported to experience severe cytokine storm, with increased serum levels of proinflammatory cytokines like interleukin (IL)-1, IL-6, and tumor necrosis factor (TNF)-α [14], which might promote neuroinflammation and neurodegeneration [15, 16]. The proinflammatory cytokines might act directly on neurons to induce apoptosis [17]. Meanwhile, proinflammatory cytokines could cause a breach in the blood-brain barrier thereby allowing for the entry of inflammatory cells into the brain, which could induce the additional release of inflammatory and neurotoxic molecules contributing to chronic neuroinflammation and neuronal death [15]. Meanwhile, the activation of the NLRP3 inflammasome triggered during SARS-CoV-2 infection might lead to downstream tau aggregation and neurodegeneration [18]. The specific molecular mechanism by which SARS-CoV-2 activates NLRP3 inflammasomes is still unclear. A previous study has shown that SARS-CoV-2 N protein promotes the assembly of the NLRP3 inflammasome through direct interaction with NLRP3 protein [19]. In another study, the authors infected primary human CD14^+^ monocytes in vitro with SARS-CoV-2, and found that SARS-CoV-2 infection could trigger caspase-1 activation, IL-1β production, and NLRP3 puncta formation. The evidence indicates that SARS-CoV-2 infects human monocytes and triggers NLRP3 activation and a lytic form of cell death [20]. Clinically, patients with dementia were with higher severity and mortality of COVID-19 [21]. Patients with Parkinson’s disease (PD) showed worsened motor and nonmotor symptoms after being diagnosed with COVID-19 [22]. Meanwhile, several case reports described the development of acute parkinsonism, AD, or amyotrophic lateral sclerosis (ALS) following COVID-19 [23–25]. However, the observational studies might be biased by unavoidable confounding factors, and cannot determine causation. Therefore, whether COVID-19 triggers neurodegeneration is still elusive.

In this context, we performed a two-sample Mendelian randomization (MR) analysis to explore the causal role of COVID-19 in the risk of neurodegenerative disorders. The MR approach is less susceptible to reverse causation or confounding factors which may distort the interpretations of conventional observational studies. As a result, we found that COVID-19 was causally associated with higher risk of AD.

## Methods

### Datasets

We obtained GWAS summary statistics of the susceptibility, severity, and hospitalization of COVID-19 from the COVID-19 Host Genetics Initiative [26] (https://www.covid19hg.org/, Release 6). The COVID-19 infection was defined as a positive SARS-CoV-2 infection (e.g., RNA RT-PCR or serology test), electronic health record evidence or self-reported infection from the patients. The susceptibility phenotype compared COVID-19 patients with population controls free of COVID-19 (*N*_case_ = 112,612, *N*_control_ = 2,474,079). The hospitalization phenotype was to compare patients with COVID-19 who were hospitalized and controls who were not admitted to hospitals due to COVID-19, or who were free of COVID-19 (*N*_case_ = 24,274, *N*_control_ = 2,061,529). The severity phenotype was obtained between hospitalized individuals with COVID-19 who died or required respiratory support, and controls who were without severe COVID-19, or who were free of COVID-19 (*N*_case_ = 8779, *N*_control_ = 1,001,875). Details of the summary data from all GWAS were listed in Supplementary Table 1. Single nucleotide polymorphisms (SNP) that passed the genome-wide significance threshold (*P* < 5E–08) were chosen as instrumental variants, which were then clumped based on the 1000 Genomes Project linkage disequilibrium (LD) structure. Index SNPs (*R*^2^ < 0.001 with any other associated SNP within 10 Mb) with the minimum P value were kept.

We analyzed six common neurodegenerative disorders as outcomes, including Alzheimer’s disease (AD) (*N* = 455,258) [27], Parkinson’s disease (PD) (*N* = 482,730) [28], ALS (*N* = 80,610) [29], multiple sclerosis (*N* = 115,803) [30], frontotemporal dementia (*N* = 12,928) [31], and Lewy body dementia (*N* = 6618) [32] based on summary statistics from previous GWAS with large sample size. The study design like the collection of samples, quality control procedures, and imputation methods have been described in the original publications. Harmonization was undertaken to rule out strand mismatches and ensure alignment of SNP effect sizes. The study was approved by West China Hospital, Sichuan University.

### Genetic correlation

We estimated the genetic correlation between COVID-19 and each neurodegenerative disorder using GNOVA with default parameters [33]. GNOVA estimates genetic covariance with the genetic variants summary data shared between two GWAS, and then calculates the genetic correlation based on genetic covariance and variant-based heritabilities. The European dataset from the 1000 Genomes Project was used as reference data. A *P* value below 2.78E–03 (0.05/18) was considered statistically significant after the Bonferroni correction.

### Mendelian randomization analysis

We hypothesized that COVID-19 as a risk factor could causally influence the risk of neurodegenerative disorders, and the following assumptions were satisfied: the genetic variants as instrumental variables are associated with COVID-19; the genetic variants are not associated with any confounders; the genetic variants are associated with risk of neurodegenerative disorders through COVID-19 (namely horizontal pleiotropy should not be present) (Supplementary Fig. 1).

To evaluate the causative effect of COVID-19 on the risk of neurodegenerative disorders, we performed two-sample MR analysis using the random effects inverse variance weighted (IVW) method, which is most widely used in MR studies and could provide robust causal estimates under the absence of directional pleiotropy. A *P* value below 2.78E−03 (0.05/18) was considered statistically significant after the Bonferroni correction. We further verified the results using the weighted median method, which generally has greater power with a positive causal effect, particularly as the proportion of invalid instrumental variables increases [34]. In addition, we conducted comprehensive sensitivity analyses to estimate potential violations of the model assumptions in the MR analysis. We conducted Mendelian randomization pleiotropy residual sum and outlier (MR-PRESSO) analysis and leave-one-out analysis to detect outlier instrumental variables [35]. Outlier instrumental variables identified by the MR-PRESSO outlier test were removed step-by-step to reduce the effect of horizontal pleiotropy. Cochran’s Q test was executed to check the heterogeneity across the individual causal effects. MR-Egger regression was performed to evaluate the directional pleiotropy of instrumental variables [36]. To evaluate the strongness of each instrumental variable, we computed the F-statistic of each SNP [37]. The statistical power was calculated using an online tool at http://cnsgenomics.com/shiny/mRnd/ [38]. The statistical analyses were conducted using the R package TwoSampleMR 0.5.5 [39].

## Results

**Fig. 1:**
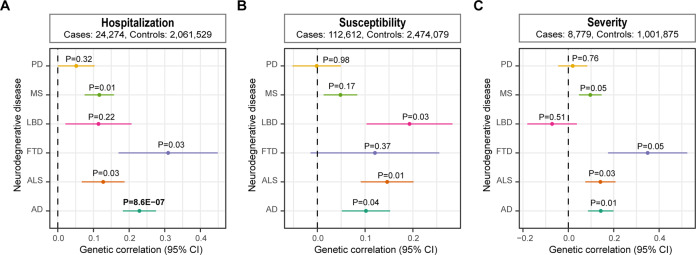
Forest plot showing results from the genetic correlation analysis. Genetic correlation between **A** hospitalization, **B** susceptibility, **C** severity of COVID-19 and neurodegenerative disorders. Error bars indicate 95% confidence intervals. Bold *P* value denotes statistical significance after the Bonferroni correction.

We first estimated the genetic correlation between COVID-19 and each neurodegenerative disorder. We detected a significant positive genetic correlation between hospitalization of COVID-19 and AD after the Bonferroni correction (genetic correlation: 0.23, *P* = 8.36E−07) (Fig. 1).

**Fig. 2:**
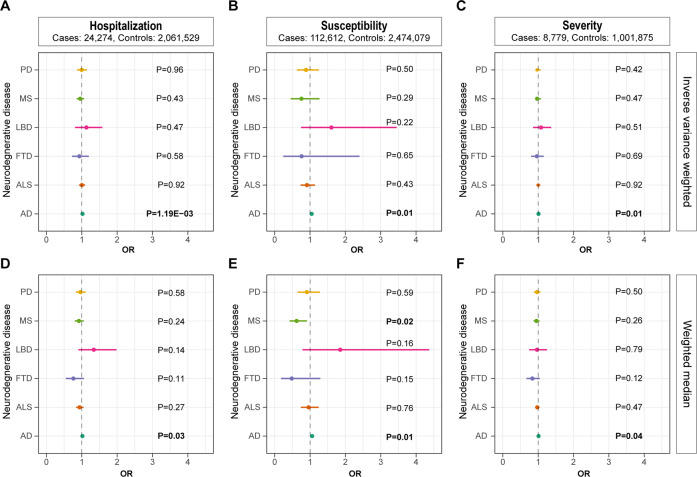
Forest plot showing results from the Mendelian randomization analysis. Results from the Mendelian randomization (MR) analysis to evaluate causal role of **A** hospitalization, **B** susceptibility, and **C** severity of COVID-19 in neurodegenerative disorders using the inverse variance weighted method. Results from the MR analysis to evaluate potential causal role of **D** hospitalization, **E** susceptibility, and **F** severity of COVID-19 in neurodegenerative disorders using the weighted median method. Estimates are per 1 standard deviation (SD) increase in the trait. Bold *P* value denotes nominal association (*P* < 0.05).

Furthermore, we analyzed the role of COVID-19 in the risk of each neurodegenerative disorder via the two-sample MR approach. Results showed that hospitalization of COVID-19 was significantly associated with a higher risk of AD (OR: 1.02, 95% CI: 1.01−1.03, P: 1.19E−03) after the Bonferroni correction (Fig. 2A). Nominal association was identified using the weighted median method (OR: 1.02, 95% CI: 1.00−1.04, *P*: 0.03) (Fig. 2D and Supplementary Fig. 2). Consistently, susceptibility and severity of COVID-19 were nominally associated with higher risk of AD using both the IVW and the weighted median methods (Fig. 2B, C, E, F and Supplementary Fig. 2), further strengthening the hypothesis that COVID-19 could increase the risk of AD.

Next, we performed extensive sensitivity analyses to validate the causal association between COVID-19 and the risk of neurodegenerative disorders. The Cochran’s Q test did not detect the heterogeneity of effects across the instrumental variables (Table 1). The F statistics of all the instrument variables were above 10 (ranging from 29 to 399), indicating the absence of weakness in the selected instruments. No apparent horizontal pleiotropy was observed as the intercept of MR-Egger was not significantly deviated from zero (Table 1). Meanwhile, no potential instrumental outlier was detected at the nominal significance level of 0.05 by the MR-PRESSO analysis (Table 1). The leave-one-out results suggest that the causal effect was not driven by a single instrumental variable (Supplementary Figs. 2–7).

**Table 1:** Heterogeneity and horizontal pleiotropy analyses between COVID-19 and neurodegenerative disorders.

Exposure trait	Outcome trait	Heterogeneity			Horizontal pleiotropy			MR-PRESSO *P* value	Variance	Beta
		IVW Q	IVW Q df	IVW P value	Egger intercept	SE	*P* value			
COVID-19 susceptibility	AD	6.78	6	0.24	−1.61E−03	2.45E−03	0.54	0.352	3.70E−04	0.49
	ALS	1.83	5	0.87	−2.08E−03	0.015	0.89	0.867	3.55E−04	0.90
	FTD	8.39	4	0.08	0.12	0.07	0.16	0.108	2.24E−04	1.61
	LBD	1.97	4	0.74	−0.05	0.05	0.42	0.675	2.00E−04	2.12
	MS	6.87	3	0.08	0.06	0.04	0.25	0.065	2.08E−04	1.00
	PD	9.2	6	0.16	−0.03	0.02	0.34	0.296	3.70E−04	0.62
COVID-19 hospitalization	AD	14.95	14	0.38	1.21E−03	1.61E−03	0.47	0.476	5.41E−04	0.42
	ALS	14.79	13	0.32	0.02	0.01	0.1	0.215	5.18E−04	0.78
	FTD	9.47	9	0.4	0.04	0.03	0.26	0.278	4.58E−04	1.42
	LBD	14.77	10	0.14	0.1	0.07	0.21	0.166	2.84E−04	2.02
	MS	13.42	11	0.27	0.01	0.01	0.29	0.272	4.99E−04	0.68
	PD	19.21	12	0.08	0.01	0.02	0.43	0.132	4.92E−04	0.55
COVID-19 severity	AD	11.42	12	0.49	5.03E−04	1.72E−03	0.78	0.572	9.05E−04	0.34
	ALS	13.93	13	0.31	0.02	0.01	0.11	0.219	9.05E−04	0.63
	FTD	8.14	7	0.32	0.04	0.04	0.3	0.178	7.76E−04	1.25
	LBD	16.65	9	0.06	0.03	0.06	0.68	0.056	4.57E−04	1.86
	MS	14.77	10	0.14	0.02	0.02	0.36	0.192	8.28E−04	0.54
	PD	12.99	11	0.29	−4.62E−03	0.02	0.98	0.362	8.73E−04	0.44

*IVW* Inverse variance weighted; *Q* Cochran’s Q test estimate, *df* Cochran’s Q test degrees of freedom, *SE* standard error, *AD* Alzheimer’s disease, *ALS* amyotrophic lateral sclerosis, *FTD* frontotemporal dementia, *LBD* Lewy body dementia, *MS* multiple sclerosis, *PD* Parkinson’s disease. Variance means the variance explained by instrumental variables for the exposure trait. Beta means effect size which can be detected with the power of 0.8 given the sample size, proportion of cases, and variance explained by instrumental variable for each Mendelian randomization analysis.

## Discussion

Previous clinical studies have suggested that COVID-19 may trigger clinical manifestations of neurodegenerative disorders. Functional exploration of SARS-CoV-2 in the brain also reinforced such hypothesis. However, as most neurodegenerative disorders are late-onset and slowly progressive, current epidemiological studies might not detect the effect to an observable extent. Meanwhile, unmeasured confounding factors in clinical studies can potentially bias the association evidence, as is a common criticism inherent to observational studies. Therefore, we investigated the causative role of COVID-19 in the risk of neurodegenerative disorders using the MR approach. The results showed that COVID-19 could increase the risk of AD. Such association was detected for susceptibility, hospitalization, and severity of COVID-19. These findings provided a better understanding of the role of COVID-19 in the risk of neurodegenerative disorders, and had clinical implications for patients, clinicians and researchers.

The two previous noteworthy outbreaks caused by coronaviruses, namely severe acute respiratory syndrome (SARS) and the Middle East respiratory syndrome (MERS), both caused memory impairment during and after the illness [40], suggesting a potential role of COVID-19 in cognitive impairment as well. Dementia or cognitive impairment as complications of COVID-19 has already been reported frequently in retrospective cohort studies [5, 41, 42]. Around 0.67% of patients with COVID-19 and 1.74% of patients with COVID-19 admitted to ITU developed dementia within 6 months after diagnosis [5]. Similarly, another nationwide cohort study from South Korea among 306,577 adults found that the incidence of dementia among COVID-19 survivors was 1.39-fold higher (HR: 1.39, 95% CI: 1.05–1.85; *P* = 0.023). Among the subtypes of dementia, COVID-19 survivors were in higher risk of AD (HR: 1.32, 95% CI: 1.05–1.86; *P* = 0.028) and other types of dementia (HR: 2.04, 95% CI: 1.25–3.32; *P* = 0.004), but not vascular dementia (HR: 1.51, 95% CI: 0.62–3.70; *P* = 0.364) [43]. The high levels of proinflammatory cytokines, hypoxia, and direct infection into the brain by the SARS-CoV-2 might contribute to the development of cognitive impairment [44]. Previous research has shown that SARS-CoV-2 infection could activate TGF-β signaling and oxidative overload, and the neuropathological pathways causing tau hyperphosphorylation typically associated with AD were activated in COVID-19 patients [45]. The exact mechanism of how COVID-19 leads to tau phosphorylation and aggregation is still poorly understood. One possible explanation is that activation of the NLRP3 inflammasome triggered during SARS-CoV-2 infection could promote tau hyperphosphorylation [18, 46]. Meanwhile, a network-based, multimodal omics comparison of COVID-19 and neurologic complications also identified significant mechanistic overlap between AD and COVID-19, mainly centered on neuroinflammation and brain microvascular injury [47]. All these findings suggested that patients diagnosed with COVID-19 might have an acceleration of Alzheimer’s-related symptoms and pathology. From a genetic perspective, our results provided evidence for the causal role of COVID-19 in AD, though the effect size was limited. The small effect might be due to the insufficient instrumental variables, since the significant SNPs only explain a small proportion of the variance in the exposures (Table 1). Therefore, further replication based on GWAS with larger sample size was still necessary.

In contrast, we did not identify a causal association between COVID-19 and the other neurodegenerative disorders. This might be due to the differences in the pathogenesis of AD from other diseases. However, we cannot exclude the possibility that we failed to detect the association due to the insufficiency of current sample sizes. The variance explained by the instrumental variables of the exposures was moderate, which limited the power to detect weaker causal associations. With summary statistics from future GWAS with larger sample sizes, the association of COVID-19 with other neurodegenerative disorders might become significant. In addition, the hospitalization and severity phenotype of COVID-19 might be influenced by various factors like medical situations in each country, which could not be accounted for in the MR analysis. Meanwhile, there was potential population structure in the GWAS of COVID-19, since individuals from different countries were involved. However, since no great heterogeneity was detected for each instrumental variable we utilized from the original GWAS of COVID-19, the population structure should not influence the association much. Nevertheless, future studies on this topic are still warranted.

In conclusion, our results demonstrated that COVID-19 was genetically correlated to AD. Meanwhile, susceptibility, hospitalization, and severity of COVID-19 could increase the risk of AD. These findings help better understand the role of COVID-19 in neurodegenerative disorders, and will facilitate therapeutic drugs in future clinical trials to alleviate the complications of COVID-19.

## Supplementary information

Supplementary Figures

Supplementary Tables

## Data Availability

Summary statistics of COVID-19 could be downloaded from the COVID-19 Host Genetics Initiative (https://www.covid19hg.org/, release 6). Summary statistics of each neurodegenerative disorder could be found in the original publication. The datasets generated during the analysis were in the supplementary materials.

## Code availability

The code and algorithm used to generate results in this study are available from the corresponding authors upon reasonable request.
