# Supplementary material for: COVID-19 and risk of neurodegenerative disorders: A Mendelian randomization study
Source: Transl Psychiatry. 2022 Jul 14;12:283. doi: 10.1038/s41398-022-02052-3 (PMC9281279; doi:10.1038/s41398-022-02052-3)

Supplementary Fig. 1. Assumptions in Mendelian randomization analysis

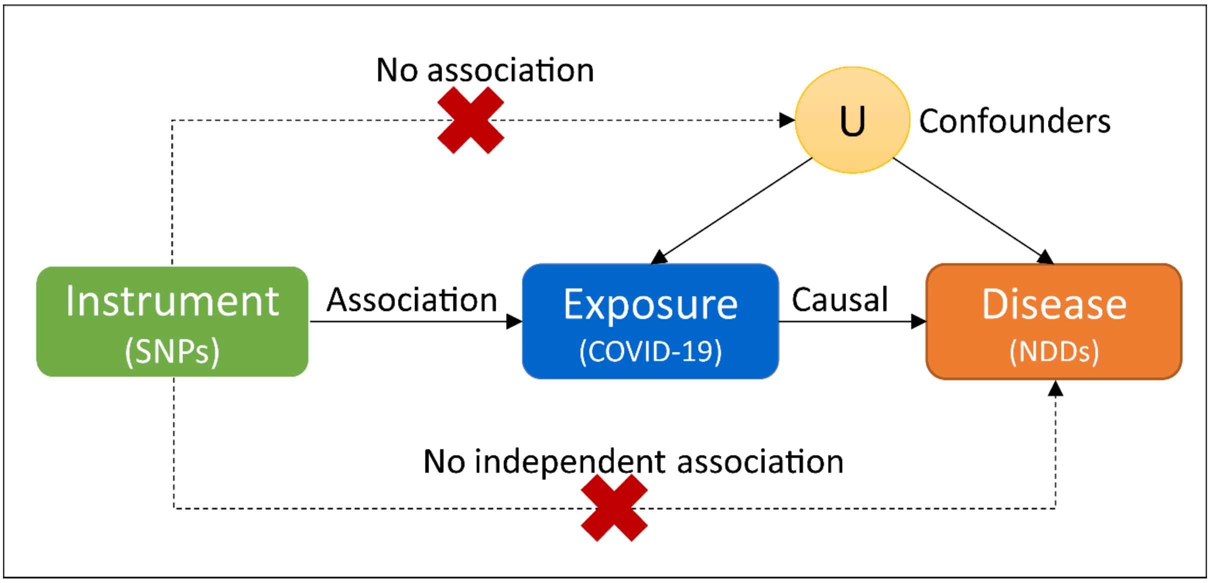

Broken lines represent potential pleiotropic or direct causal effects that would violate Mendelian randomization assumptions. Assumption 1: Genetic variants are associated with the COVID-19; Assumption 2: Genetic variants are not associated with confounders; Assumption 3: Genetic variants influence risk of neurodegenerative disorders (NDDs) only through the exposure.

Supplementary Fig. 2. Mendelian randomization analysis results for COVID-19 on risk of Alzheimer's disease.

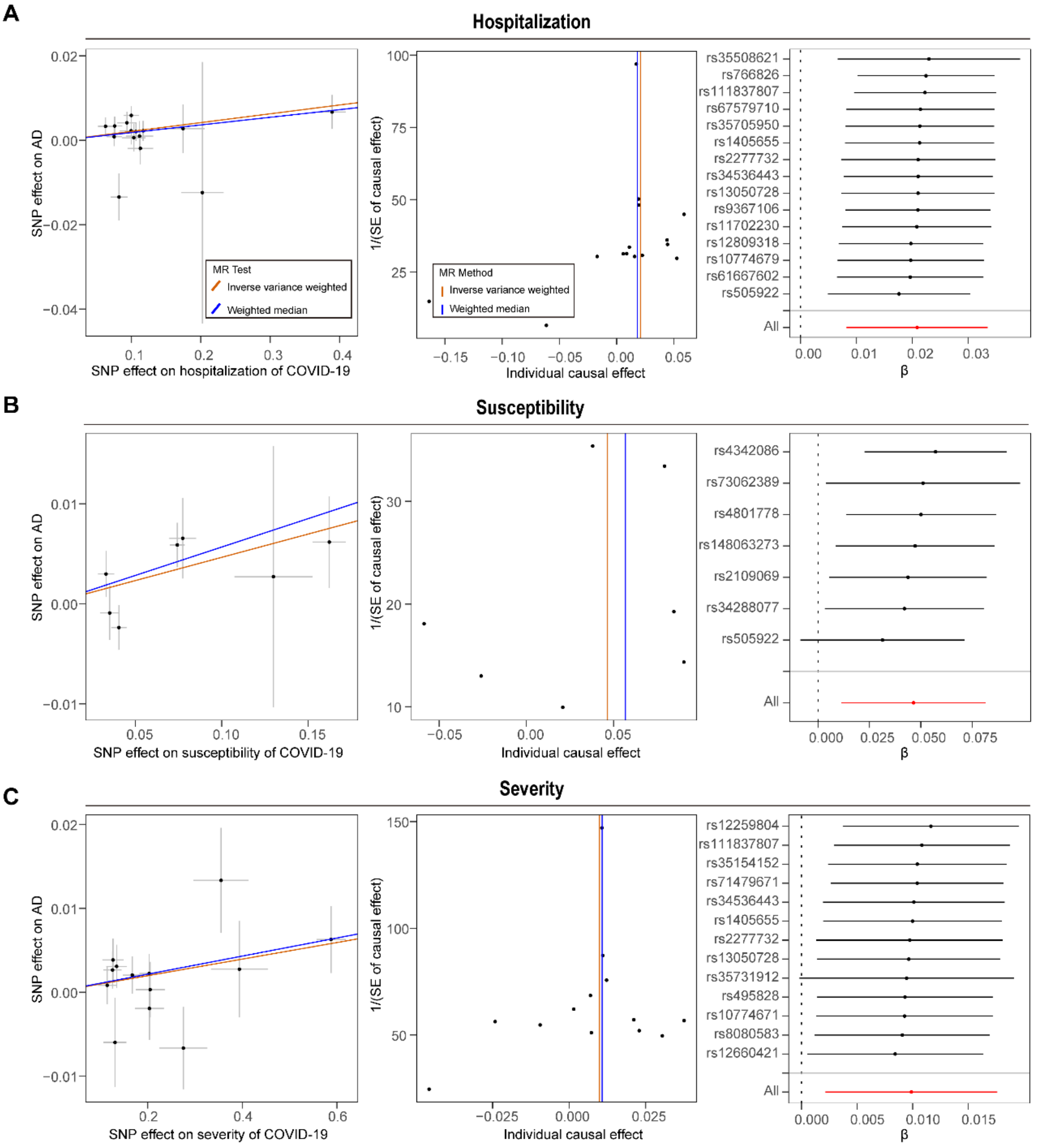

Column 1: Scatter plot of single nucleotide polymorphism (SNP) potential effects on COVID-19 and Alzheimer's disease. The 95% CI for the effect size on Alzheimer's disease is shown as vertical lines, while the 95% CI for the effect size on COVID-19 is shown as horizontal lines. The slope of fitted lines represents the estimated MR effect per method. Column 2: Funnel plot for COVID-19 shows the estimation using the inverse of the standard error of the causal

estimate with each individual SNP as a tool. The vertical line represents the estimated causal effect obtained using IVW and MR-Egger methods. Column 3: Forest plot of the results of the leave-one-out sensitivity analysis, where each SNP in the instrument was iteratively removed from the instrument variables.

Supplementary Fig. 3. Mendelian randomization analysis results for COVID-19 on risk of amyotrophic lateral sclerosis.

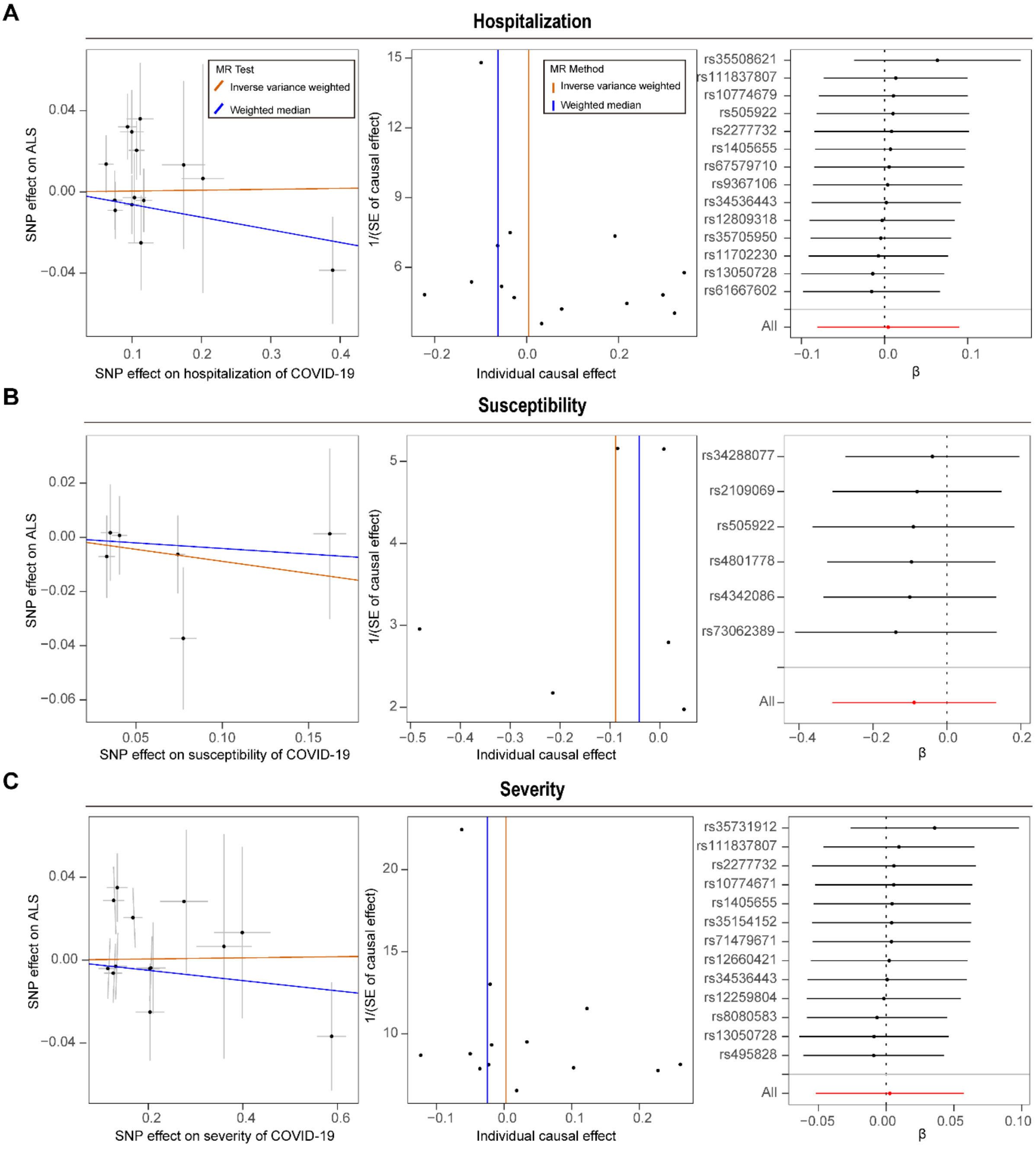

Supplementary Fig. 4. Mendelian randomization analysis results for COVID-19 on risk of frontotemporal dementia.

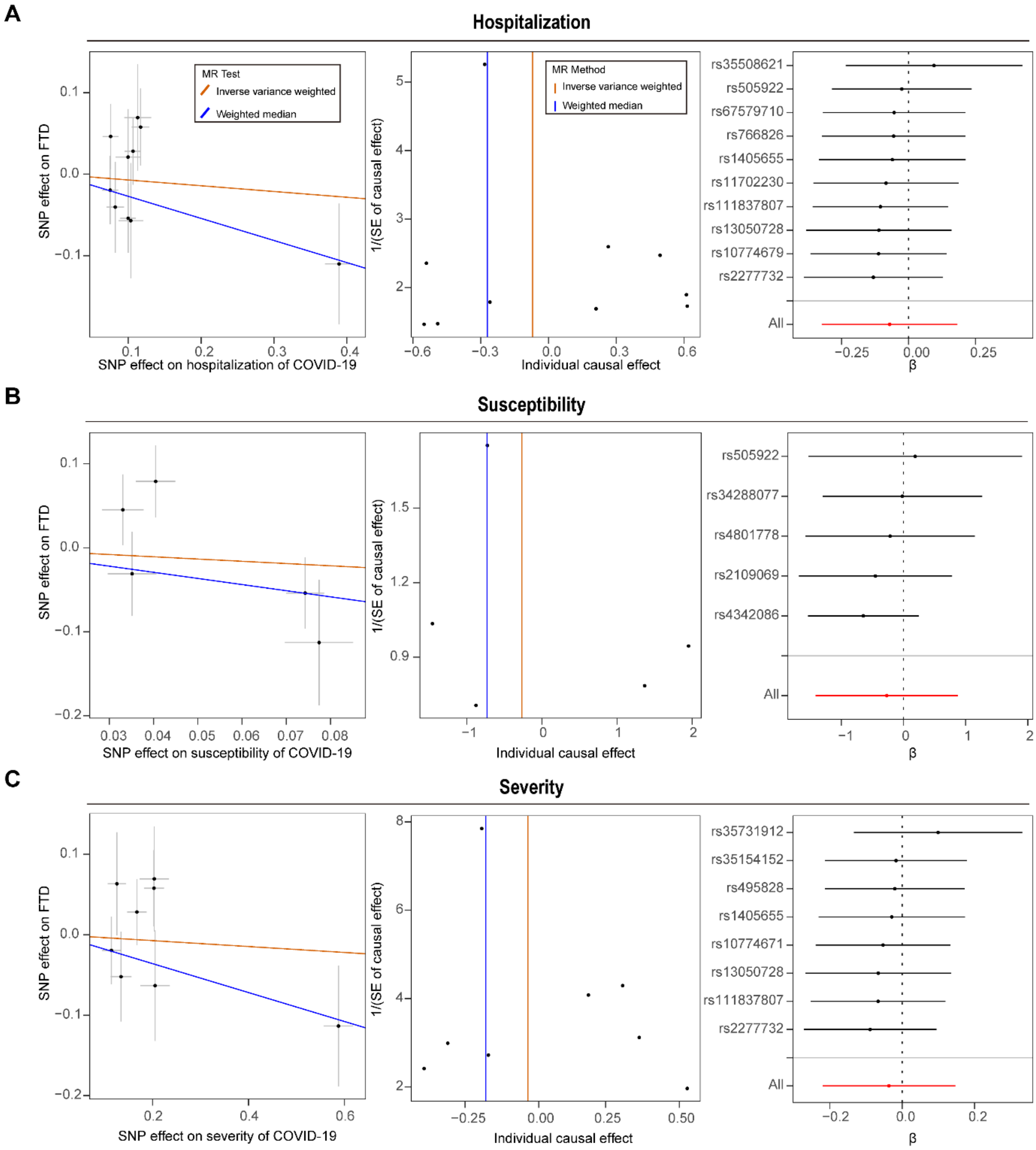

Supplementary Fig. 5. Mendelian randomization analysis results for COVID-19 on risk of Lewy body dementia.

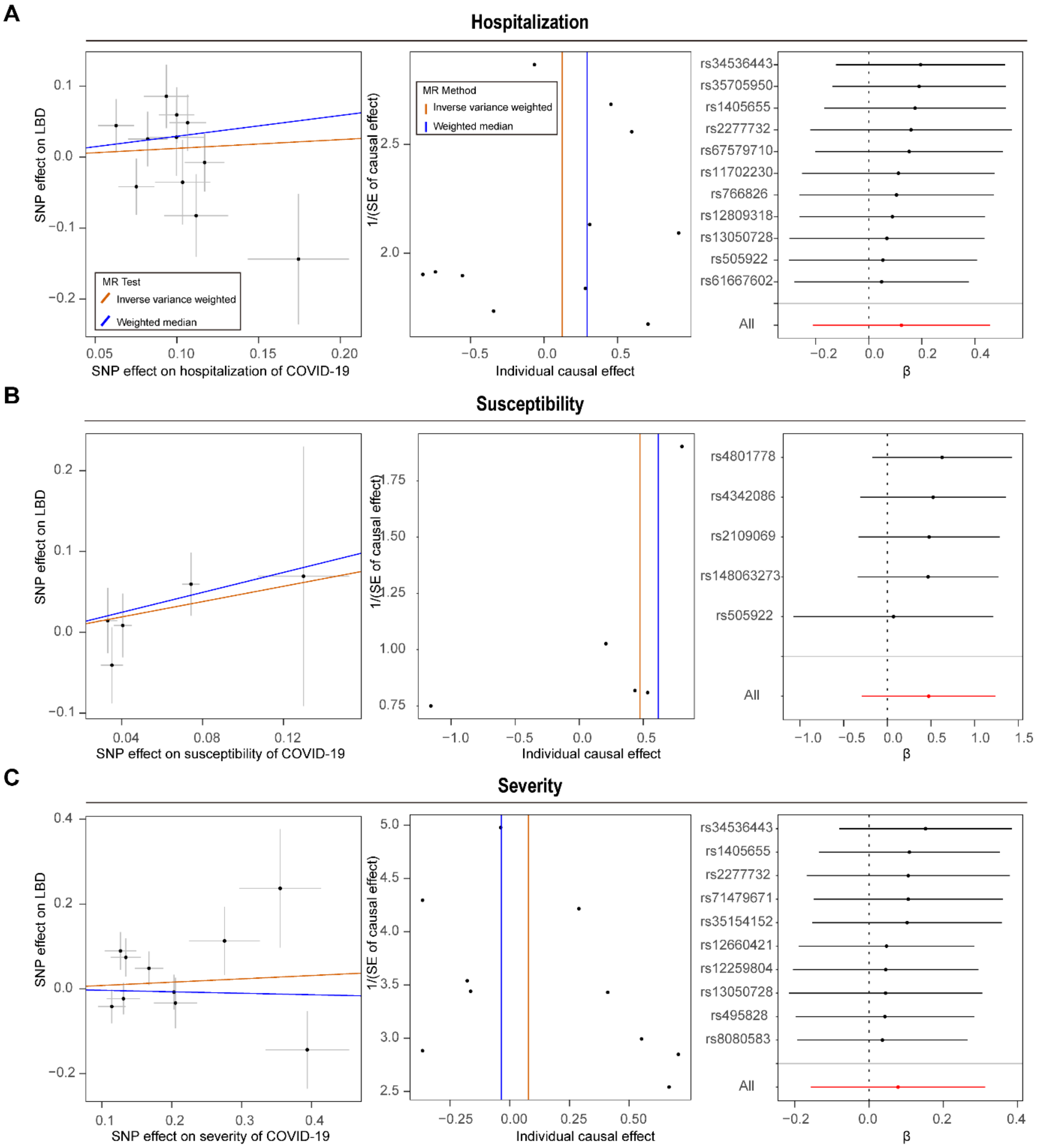

Supplementary Fig. 6. Mendelian randomization analysis results for COVID-19 on risk of multiple sclerosis.

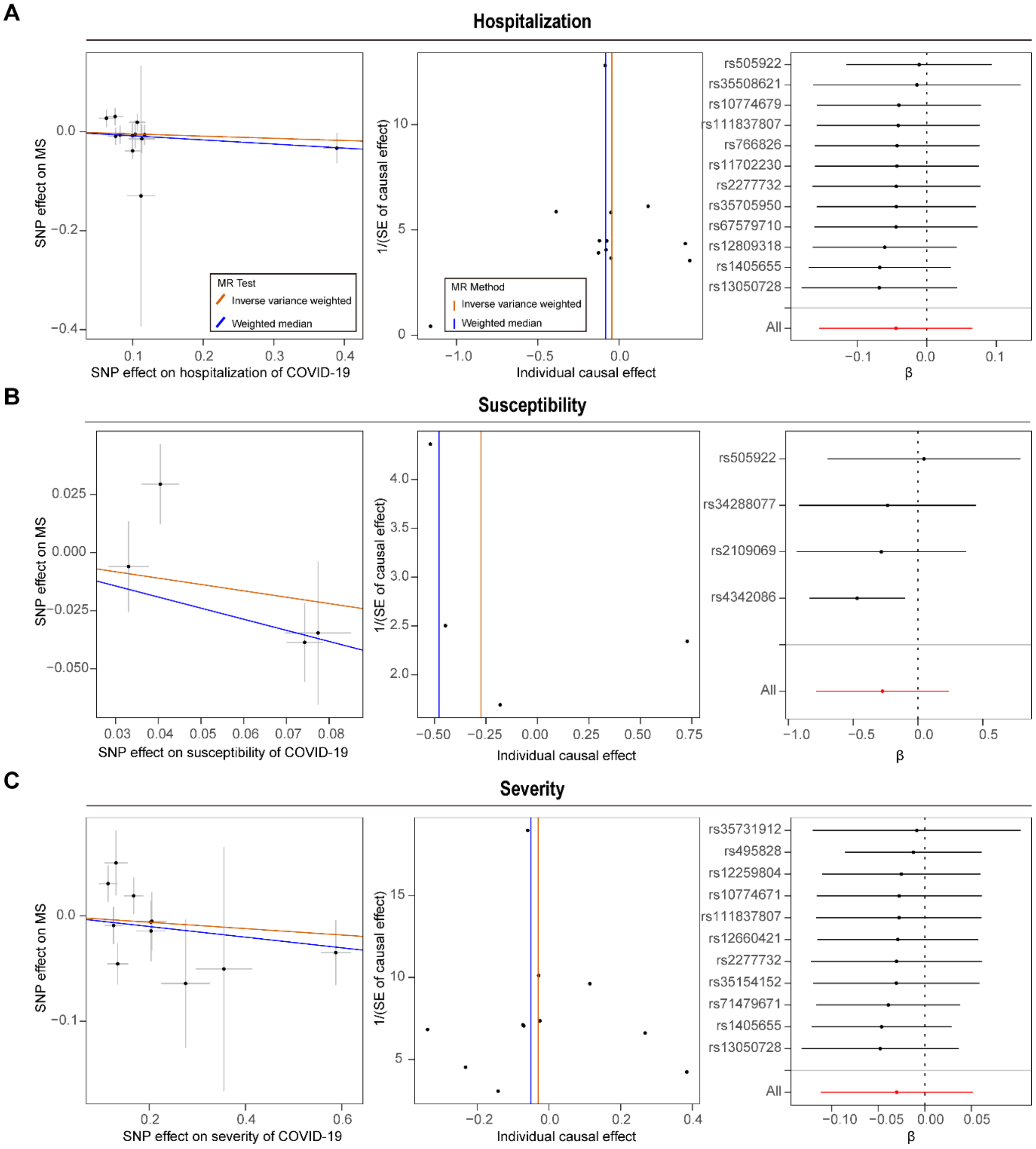

Supplementary Fig. 7. Mendelian randomization analysis results for COVID-19 on risk of Parkinson's disease.

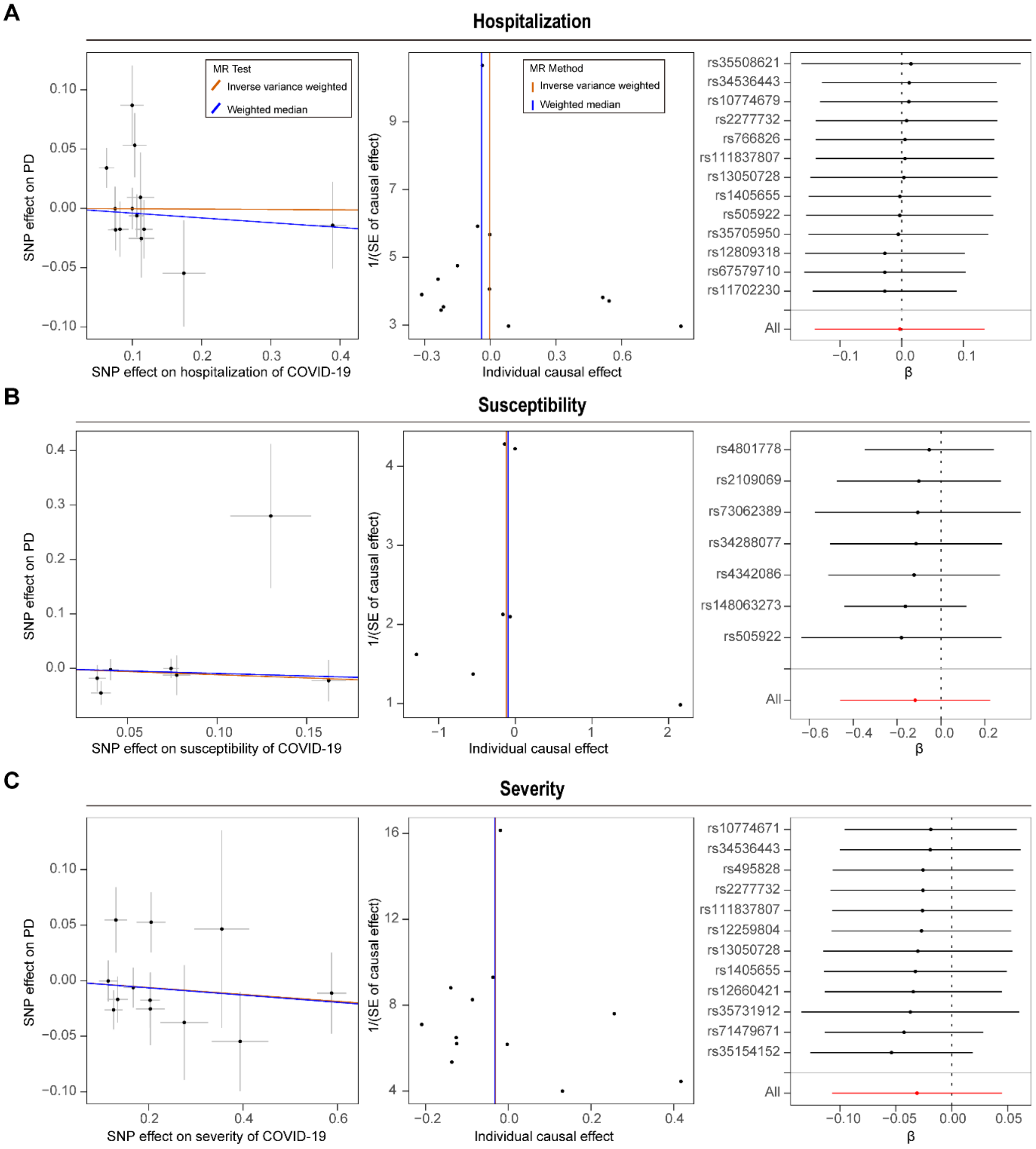

Supplement: Supplementary file 1 — Supplementary Figures [file 41398_2022_2052_MOESM1_ESM.pdf]
