# Supplementary material for: COVID-19 and risk of neurodegenerative disorders: A Mendelian randomization study
Source: Transl Psychiatry. 2022 Jul 14;12:283. doi: 10.1038/s41398-022-02052-3 (PMC9281279; doi:10.1038/s41398-022-02052-3)
Supplement: Supplementary file 2 — Supplementary Tables [file 41398_2022_2052_MOESM2_ESM.pdf]

Supplementary Table 1. Summary data from all GWAS used in current study.

Disease	abbreviation	Cases	Controls	Ethnics	Number of SNPs	PMID
susceptibility of COVID-19	n.d.	112,612	2,474,079	Mixed	12,222,969	32404885
hospitalization of COVID-19	n.d.	24,274	2,061,529	Mixed	11,810,332	32404885
severity of COVID-19	n.d.	8,779	1,001,875	Mixed	8,141,091	32404885
Parkinson's disease	PD	33,674	449,056	EUR	17,510,617	31701892
Alzheimer's disease	AD	71,880	383,378	EUR	13,367,299	30617256
amyotrophic lateral sclerosis	ALS	20,806	59,804	EUR	10,031,417	29566793
multiple sclerosis	MS	47,429	68,374	EUR	6,304,358	31604244
Lewy body dementia	LBD	2,591	4,027	EUR	7,654,310	33589841
frontotemporal dementia	FTD	3,526	9,402	EUR	6,026,384	24943344

EUR, European; Mixed ethnics denotes individuals from different ancestry like Asian, European, American and African; SNP, single nucleotide polymorphism; GWAS, genome-wide association study; PMID, PubMed ID; n.d., not defined.
